# Comparative Effects of Premedication with Ibuprofen and Essential Oil of Urtica Dioica on Post-Endodontic Pain: Clinical Trial

**DOI:** 10.30476/dentjods.2024.100104.2193

**Published:** 2024-12-01

**Authors:** Parham Elahinia, Armita Vali Sichani, Asana Vali Sichani, Navid Yaraghi, Afsaneh Yegdaneh, Zahra Khosravani

**Affiliations:** 1 School of Dentistry, Isfahan University of Medical Sciences, Isfahan, Iran; 2 Dental Materials Research Center, Dental Research Institute, Dept. of Endodontics, Isfahan, Iran; 3 School of Dental Medicine, Boston University Henry. M. Goldman, USA; 4 Orthodontic in Private Clinic, Isfahan, Iran; 5 Dept. of Pharmacognosy, School of Pharmacy and Pharmaceutical Sciences, Pharmaceutical Sciences Research Center, Isfahan University of Medical Sciences, Isfahan, Iran; 6 Dep. of Endodontics, School of Dentistry, Bushehr University of Medical Sciences, Bushehr, Iran

**Keywords:** Pain, Endodontics, Stinging Nettle, Brufen, Alternative medicine, Complementary therapies, Urtica dioica

## Abstract

**Statement of the Problem:**

Considering side effects of non-steroidal anti-inflammatory drugs (NSAIDs), herbal medicine including *Urtica dioica* might help reduce the pain

**Purpose:**

The present study aimed to compare the effects of premedication with essential oil of *Urtica dioica* and ibuprofen on post-endodontic pain.

**Materials and Method:**

The present randomized clinical trial study was conducted on 60 patients with symptomatic irreversible pulpitis of mandibular first or second molars. The patients were randomly assigned to three groups (n=20) for premedication with 400 mg ibuprofen, 400 mg essential oil of *Urtica dioica* (Netonal; Barij, Iran), and placebo. The medications were taken 30 minutes prior to the procedure. The patients’ pain score was recorded before the intervention and treatment onset (Time1 or T1), after the intervention prior the treatment onset (10 minutes after anesthetic injection) (T2), upon completion of treatment (T3), and at 8 (T4), 12 (T5), and 24 hours after endodontic treatment (T6) using a visual analog scale (VAS) and Wong-Baker FACES Pain Rating Scale (WBS), and compared. Data were analyzed by Chi-square test, one-way and repeated measures analysis of variance (ANOVA), kruskal-wallis test, and LSD post-hoc test using IBM SPSS statistics version 21 with significance value of *p*< 0.05.

**Results:**

The pain score was not significantly different among the three groups at T1, T2 and T3 according to both scales (*p*> 0.05). On the other hand, significantly lower pain scores were recorded in ibuprofen and Urtica dioica groups at 8, 12, and 24 hours after treatment compared with the placebo group (*p*< 0.001). The pain score was not markedly different between the ibuprofen and *Urtica dioica* groups (*p*> 0.05).

**Conclusion:**

It seems that analgesic effect of premedication with leaf extract of *Urtica dioica* is the same as ibuprofen. Further studies are warranted to find the optimal dosage of *Urtica dioica* for widespread use.

## Introduction

Pulpitis is defined as inflammation of the pulp tissue. Considering the viability of pulp tissue and absence of complete necrosis, teeth with pulpitis respond to cold and hot stimuli. As a result, such teeth should either undergo root canal treatment or should be extracted [ 1 - 2 ]. The prevalence of post-endodontic pain is as high as 70% [ 3 ]. It is a common complication of endodontic treatment and can cause hesitation about visiting a dentist [ 4 ]. Postoperative endodontic pain is a flare-up characterized by pain, swelling, or both, which occurs shortly (within a couple of hours) after endodontic treatment [ 3 ]. Preoperative and procedural factors such as mechanical instrumentation, microbial effects, chemical stimulations, and intracanal medicaments may irritate or injure the peri-radicular tissue and lead to post-endodontic pain [ 5 ]. Irrigation solution and irrigation of the teeth for cleaning the surrounding tissues stimulate the periradicular and cause pain in post operation. These methods include the activation of the solution using gutta-percha cones, negative pressure, canal brushes, laser systems, and sound/ultrasonic devices placed along the channel [ 6 ]. Moreover, instrument technique plays an important role in shaping and cleaning the root canal system and affects the occurrence and intensity of postoperative pain [ 7 ].

Tissue damage results in activation of inflammatory processes and pain receptors through inflammatory mediators and especially prostaglandins. Prostaglandins activate the sensory periapical nociceptors, increase vascular permeability and chemotactic activity, and elevate the sensitivity of pain receptors to other activated inflammatory mediators [ 8 ]. 

Post-endodontic pain is a significant clinical problem for both patients and dental clinicians [ 9 ]. A systematic review revealed that premedication in patients with reversible pulpitis would decrease postoperative pain [ 10 ] Literature suggests administration of one dose of an anti-inflammatory medication preoperatively to control inflammatory mediators and prevent post-endodontic complications and administration of another dose after the procedure [ 8 ]. 

Non-steroidal anti-inflammatory drugs (NSAIDs) are major analgesics that inhibit the activity of cyclooxygenase enzyme, prevent the synthesis of new prostaglandin molecules, and exert analgesic and anti-inflammatory effects [ 11 ]. Among NSAIDs, ibuprofen is commonly prescribed due to its high safety margin and low cost. It has excellent efficacy for reduction of post-endodontic pain and inflammation with minimal side effects [ 12 - 13 ]. The optimal analgesic efficacy of ibuprofen for alleviation of post-endodontic pain has been reported in the literature [ 14 ]. However, long-term use of NSAIDs, such as ibuprofen, can have side effects such as dizzyness, constipation and gastrointestinal problems [ 15 ]. 

Medicinal plants have long been used in Iran for prevention and treatment of numerous diseases [ 16 ]. Urtica dioica (U. dioica) or stinging nettle is a member of the Urticaceae family, which is native to Eurasia [ 17 - 18 ]. Its Persian name is “Aragh Gazaneh” meaning stinging [ 16 ]. U. dioica is abundantly found in Asia, Europe, North Africa, and North America [ 18 ]. Fresh leaves and aerial flowering parts of U. dioica and Urtica urens and a combination of them have been used for pain relief [ 19 ]. Several chemical agents such as acetylcholine, histamine, serotonin-HT5, meridian, leukotrienes, and probably formic acid are present in U. dioica trachoma’s [ 20 - 21 ]. Furthermore, U. dioica contains phytochemicals such as phenolic compounds, sterol, fatty acids, alkaloids, triterpenoids, flavonoids, lignans, sesquiterpenoid, and sphingolipid [ 22 - 23 ]. U. dioica has antiviral, antimicrobial, anti-cancer, nephroprotective, hepatoprotective, cardioprotective, anti-arthritis, anti-diabetes, antioxidant, anti-endometriosis, anti-aging, anti-allergic, analgesic, and anti-inflammatory effects [ 18 , 24 - 25 ]. 

A review study reported the applications of U. dioica for treatment of abdominal pains, rheumatic pains, cough, cold, liver problems, colitis, cancer, immune system regulation, reduction of blood cholesterol and blood sugar, cardiovascular diseases, and treatment of hypertension. Moreover, U. dioica is an edible plant containing minerals, protein, chlorophyll, and fiber, and is used as a vegetable [ 25 ]. Farahpour *et al*. [ 19 ] reported that the hydroalcoholic extract of U. dioica (especially at the dosage of 100mg) had a greater anti-nociceptive effect than the control group and diclofenac in acid-induced writhing test in rats. Gorzalczany *et al*. [ 26 ] indicated that the *Urtica circularis* extract had a superior inhibitory effect on pain than indomethacin. Nonetheless, Gohari *et al*. [ 16 ] indicated the optimal efficacy of U. dioica for reduction of abdominal cramp pain after acetic acid injection in rats. Those receiving U. dioica in 400 mg dosage experienced 81% pain reduction while indomethacin caused 84% reduction in pain. However, toxicity tests indicated a better safety margin of all the solvent extracts of U. dioica with LC_50_> 1000 μg/mL each on *Artemia salina*. In studies on mice, symptoms of diarrhea and diuresis were observed at the dose of 2000 mg/kg bw [ 27 ].

The aforementioned studies indicated variable results regarding the analgesic efficacy of U. dioica in comparison with NSAIDs for non-odontogenic pains. On the other hand, search of literature by the authors yielded no study comparing the effects of U. dioica and ibuprofen on post-endodontic pain in teeth with symptomatic irreversible pulpitis. As a result, the present study was conducted to compare the effects of premedication with U. dioica and ibuprofen on post-endodontic pain in teeth with symptomatic irreversible pulpitis. Moreover, the null hypothesis was that post-endodontic pain in teeth with symptomatic irreversible pulpitis would not be significantly different with premedication with U. dioica and ibuprofen.

## Materials and Method

The present randomized clinical trial was conducted on 60 patients with symptomatic irreversible pulpitis of mandibular first or second molars at the Endodontics Department of the School of Dentistry, Isfahan University of Medical Sciences, Iran, from July 2019 to November 2019. The study protocol was registered in the Iranian Registry of Clinical Trials (IRCT202303130577 10N2).

### Trial design

A randomized double-blind, parallel-design placebo-controlled clinical trial study was conducted in which the experimental group 1 received premedication with U. dioica, the experimental group 2 received premedication with ibuprofen, and the control group received premedication with a placebo (sugar). The results were reported in accordance with the guidelines of the Consolidated Standards of Reporting Trials. 

### Participants, eligibility criteria, and settings

The inclusion criteria were patients with mandibular first or second molars with symptomatic irreversible pulpitis, spontaneous pain with a severity score of at least 30 mm according to the visual analog scale (VAS) or Wong-Baker FACES Pain Rating Scale (WBS), age between 10 to 70 years, normal radiographic anatomy of the tooth, prolonged response to electric pulp test and cold test (using a cotton roll cooled with Endo Ice), radiographic and clinical examination confirming the diagnosis of symptomatic irreversible pulpitis of mandibular first or second molars, and completion of treatment within one session.

The exclusion criteria were intake of analgesics in the past 12 hours, long-term use of medications which interfere with NSAIDs, allergy to NSAIDs or lidocaine, chronic systemic diseases (including the patients with kidney disease, chronic obstructive pulmonary disease, diabetes, moderate to severe heart diseases, coagulation diseases, cancer, and known autoimmune disease that diagnosed by a physician), pregnancy, periapical lesion or sinus tract, chronic periapical abscess, invasive periodontitis, irreparable teeth, teeth with previous endodontic treatment, and patients with more than one aching tooth. 

The sample consisted of 60 patients with mandibular first or second molars with symptomatic irreversible pulpitis presenting to the Endodontics Department who were selected by convenience sampling. 

### Instruments and data collection

The patients were completed a form, the first part including demographic information of patients (age, gender, marital status, smoking status, long-term medication intake, history of systemic diseases, allergy, or substance abuse). The second part of the form asked for the level of pain experienced by patients using a 100 mm VAS and a 0-5 wong-baker faces pain scale (WBFPS), and the patients were required to report their pain score before the intervention and treatment onset (T1), after the intervention before the treatment onset (10 minutes after anesthetic injection and after lip sign) (T2), upon completion of treatment (T3) and at 8 (T4), 12 (T5), and twenty-four hours after endodontic treatment (T6). The VAS comprised a 10cm line, with 0 indicating no pain at the left end and 10 indicating maximum imaginable pain in the right end.

### Intervention

After obtaining written informed consent from the patients, they were randomly assigned to one of the three groups of ibuprofen, U. dioica, and placebo (n=20). The patients were instructed to take the pills 30 minutes prior to endodontic treatment of the tooth with symptomatic irreversible pulpitis. 

The patients’ T1 pain score was recorded by the researcher, and the patients took their allocated intervention depending on their group allocation 30 minutes prior to the procedure. 

The patients in the ibuprofen group received 400mg ibuprofen tablet (Aria Pharmaceuticals, Iran) as premedication [ 9 ]. The patients in the U. dioica group received Barij Netonal tablet 400 mg (leaf essence of U. dioica) that was made by Barij Essence Pharmaceutical Company in Iran, as premedication. The control group received placebo capsules containing sugar as premedication. All medications were prepared under the supervision of a pharmacist. The medications were delivered to patients in capsules with the same shape, size, and color. The ibuprofen and Netonal tablets were crushed, but not ground, for encapsulation in order not to alter their chemical composition. 

Afterwards, patients in all groups received inferior alveolar nerve block by injection of two 1.8 mL cartridges of 2% lidocaine plus 1:80,000 epinephrine. After 10 minutes, the patients were asked to express their pain level (T2) using VAS and WBFPS, and the values were recorded. After access cavity preparation, the tooth was isolated with rubber dam and the working length was radiographically determined. Filing and flaring of the canals were performed by the step-back technique. Normal saline and 2% sodium hypochlorite were used for intracanal irrigation. The root canals were dried with paper points, and obturated with gutta-percha and zinc-oxide eugenol sealer by the cold lateral compaction technique 0.5 to 1mm shorter than the radiographic apex. The patients’ pain score was as well recorded after completion of endodontic treatment in all three groups. Correspondingly, the patients were requested to record their pain score at 8, 12, and 24 hours, postoperatively. Patients had the VAS and pain score with them but the researcher documented the reports in another document and the one which patient had, remained unmarked. The 8 and 12-hours pain score was asked by the phone and the 24 hours pain score was documented at the recall session.

No participant took medications including analgesics and antibiotics as routine. In case of consuming analgesics, the participants were excluded from the study. Moreover, antibiotic administration was not indicated in these patients since antibiotic administration following post-endodontic treatment of nonvital symptomatic teeth has no effect on pain intensity 24 hours after treatment [ 28 ].

### Outcomes (primary and secondary)

The main objective of the present study was to compare the effects of premedication with U. dioica and ibuprofen on post-endodontic pain in teeth with symptomatic irreversible pulpitis. Thus, post-endodontic pain was the primary outcome. There was no secondary outcome. 

### Sample size calculation

The sample size was calculated to be 20 (a total of 60) in each group assuming α=0.05, Z_(1-α/2)=1.96, study power (1-β) of 0.80, Z_(1-β=0.84) δ_1=δ_2=1.67, and d=1.5 using the following formula. 

$n=\frac{\left[{\left(z_{1-\frac{\alpha }{2}}+z_{\mathrm{1-\beta}}\right)}^{2}]\times (\delta_{1}^{2}+\delta_{2}^{2}\right)}{d^{2}}$

In other words, with a sample size of 20 patients, there was a possibility of 0.80 to find a minimum difference of 1.5 units between the mean VAS pain scores of the two groups at alpha=0.05. 

### Interim analyses and stopping guidelines

No interim analyses were performed, and no stopping guidelines were established. 

### Randomization

The patients were randomly assigned to three groups by block randomization using Random Allocation software with 10 blocks of 6 cells. Upon admission, each participant was allocated to one cell in one block, and assigned to one of the three groups. 

### Blinding

The patients and dental clinician were not aware of the group allocations. The medications were delivered to patients in identical capsules. The control group received placebo capsules containing sugar. 

### Ethical considerations

The study protocol was approved by the Ethics Committee of Isfahan University of Medical Sciences (IR.MUI.RESEARCH.REC.1398.214). Written informed consent was signed by the patients. The patients were not deprived of routine treatments. All methods were conducted in accordance with the relevant guidelines and regulations in accordance with the *Declaration of Helsinki*.

### Statistical analysis

Data were analyzed using IBM SPSS, Inc version 21. Descriptive data were reported as mean, standard deviation, frequency, and percentage. Data were analyzed by the Chi-square test, one-way and repeated measures ANOVA, Kruskal-Wallis test, and LSD post-hoc test. *p*<0.05 was considered statistically significant. 

## Results

### Participant flow

**Figure 1:**
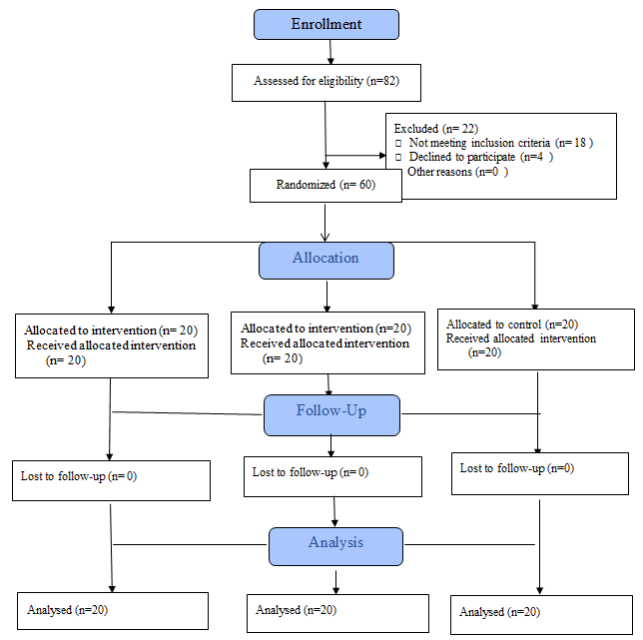
CONSORT flow diagram of patient selection and allocation

The sample consisted of 60 patients in three groups of ibuprofen, U. dioica, and placebo (n=20). Table 1 presents the demographic characteristics of the participants. The age range of patients was 25 to 59 years in the placebo group, 11 to 67 years in the ibuprofen group, and 16 to 68 years in the U. dioica group. The mean age was not significantly different among the three groups (*p*> 0.05). The number of males and females in the placebo and ibuprofen groups were the same. However, in the U. dioica group, males had a higher frequency. The Chi-square test found no marked difference in gender distribution among the three groups (*p*= 0.76). The three groups had no significant difference in marital status, level of education, history of medication intake, and history of systemic diseases either (*p*> 0.05). There were no dropouts. Figure 1 shows the CONSORT flow diagram of patient selection and allocation.

**Table 1:** Demographic characteristics of the participants

Variable		Ibuprofen	U. dioica	Placebo	*p* Value
Age Mean (SD)		39.1(10.5)	38.9 (18.2)	39.1 (10.5)	0.97^a^
Sex N (%)	Male	10(50)	12 (60)	10(50)	0.76^b^
	Female	10(50)	8(40)	10(50)	
Marital status N(%)	Married	10(50)	10 (50)	13(65)	0.23^c^
	Single	10(50)	10 (50)	7(35)	
Education N (%)	Elementary	4(20)	4(20)	5(25)	0.99^a^
	High-school Diploma	6(30)	7(35)	5(25)	
	Academic	10(50)	9(45)	10(50)	
Medication intake		8(40)	7(35)	4(20)	0.37^b^
Systemic diseases		6(30)	8(40)	4(20)	0.39^b^

a Kruskal-Wallis test

b Chi-square

c Likelihood ratio Chi-square

### Harms

No patients were harmed during the study.

### Subgroup analyses

Tables 2 and 3 show the VAS and WBFPS pain scores of patients in the three groups at different time points. Repeated measures ANOVA indicated that the interaction effect of time and group on VAS and WBFPS pain scores was significant (*p*< 0.001). Thus, one-way ANOVA was applied to compare the VAS and WBFPS pain scores among the three groups at each time point. In case of presence of a significant difference among the groups, pairwise comparisons were carried out by the LSD post-hoc test. At T1 (before the intervention and treatment onset), T2 [after the intervention and before the treatment onset (10 minutes after anesthetic injection)] and T3 (upon completion of treatment), no significant difference was found among the three groups neither in VAS nor in WBFPS pain score. However, at 8, 12, and 24 hours after the intervention, significant differences were found among the three groups in both VAS and WBFPS pain scores (*p*< 0.05). 

**Table 2:** Mean VAS pain score of patients in the three groups at different time points

Time	Ibuprofen Mean (SD)	U. dioica Mean (SD)	Placebo Mean (SD)	F	*p Value
Pre intervention and treatment	7.2 (0.5)	8.2 (0.4)	7.8 (0.5)	1.13	0.330
Post intervention and pre treatment	6.8 (0.5)	7.2 (0.6)	7.6 (0.5)	0.54	0.586
Post treatment	2.8 (0.7)	1.3 (0.4)	3.4 (0.7)	2.92	0.062
8 h after treatment	2.2 (0.5)	1.8 (0.6)	4.5 (0.6)	6.66	0.003
12 h after treatment	0.9 (0.3)	1.5 (0.5)	5.7 (0.8)	21.99	0.0001
24 h after treatment	0.6 (0.2)	1.3 (0.5)	5.6 (0.8)	23.38	0.0001

* ANOVA: Analysis of Variance; SD: Standard Deviation

**Table 3:** Mean wong-baker faces pain scale (WBFPS) pain score of patients in the three groups at different time points

Time	Ibuprofen Mean (SD)	U. dioica Mean (SD)	Placebo Mean (SD)	F	* *p* Value
Pre intervention and treatment	3.7(0.3)	4.1(0.2)	4.1(0.2)	0.71	0.494
Post intervention and pre treatment	3.4(0.3)	3.5(0.3)	3.7(0.2)	0.32	0.724
Post treatment	1.4(0.4)	0.7(0.2)	1.6(0.4)	2.69	0.076
8 h after treatment	1.1(0.2)	1(0.3)	2.4(0.3)	8.08	0.001
12 h after treatment	0.4(0.1)	0.7(0.3)	3(0.4)		0.0001
24 h after treatment	0.4(0.1)	0.7(0.3)	2.8(0.4)		0.0001

* ANOVA: Analysis of Variance; SD: Standard Deviation

The post-hoc test indicated that at 8 hours postoperatively, both the VAS (*p*= 0.005) and WBFPS (*p*= 0.001) pain scores in the ibuprofen group were significantly lower than the corresponding values in the placebo group. Moreover, at 8 hours postoperatively, both U. dioica group values were significantly lower than the corresponding values in the placebo group. On the other hand, the difference between the ibuprofen and U. dioica groups was not significant neither in VAS nor in WB-FPS pain score (*p*> 0.05). At 12 and 24 hours post operatively, the VAS (*p*= 0.0001) and WBFPS (*p*= 0.0001) pain scores in both the ibuprofen group and the U. dioica group (both *p*= 0.0001) were significantly lower than the corresponding values in the placebo group. Nonethe less, the difference between the ibuprofen and U. dioica groups was not significant neither in VAS nor in WBFPS pain score (*p*> 0.05). 

**Figure 2:**
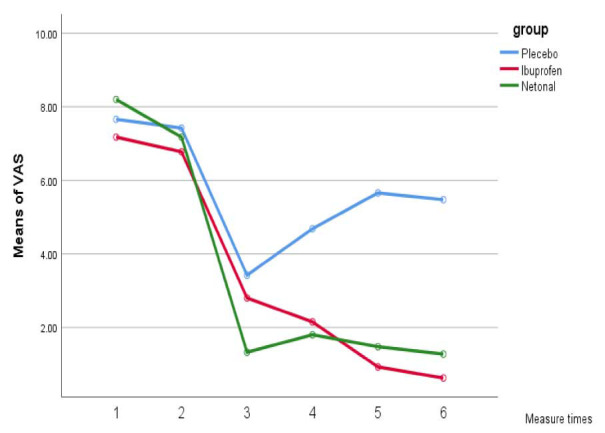
Mean visual analog scale (VAS) pain score in the three groups

**Figure 3:**
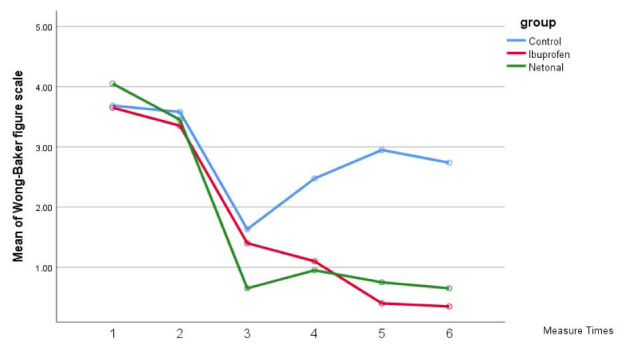
Mean wong-baker faces pain scale (WBFPS) score in the three groups

The within-group comparison of pain scores over time revealed a descending trend from T1 to T6 in both VAS and WBFPS pain scores in U. dioica and ibuprofen groups (*p*= 0.001); however, in the control group, both VAS and WBFPS pain scores had an ascending trend from 8 to 12, and 24 hours, postoperatively (*p*= 0.001) (Figures 2-3).

## Discussion

The present study compared the effects of premedicaltion with U. dioica and ibuprofen on post-endodontic pain in teeth with symptomatic irreversible pulpitis. To the best of the authors’ knowledge, the present study appears to be the first to assess the effect of U. dioica in comparison with ibuprofen on post-endodontic pain in teeth with symptomatic irreversible pulpitis. The results indicated significantly lower pain scores in the ibuprofen and U. dioica groups than the placebo group at 8, 12, and 24 hours after endodontic treatment. The difference in pain scores between the U. dioica and ibuprofen groups was not significant at any time point. 

In the present study, the ibuprofen group indicated significantly lower pain scores at 8, 12 and 24 hours post-treatment than the placebo group. Ibuprofen has a long history of use as an analgesic for prevention or management of endodontic pain. The optimal efficacy of ibuprofen for endodontic pain control has been well confirmed in the literature. Arsalan *et al*. [ 9 ] displayed that intake of one dose of 200 mg ibuprofen and 20 mg tenoxicam significantly decreased post-endodontic pain at 6 hours after the procedure in both the intervention groups, compared with the placebo, but no marked difference was found between the two intervention groups. At 12, 24, and 36 hours, however, the difference in pain score among the three groups was not significant in their study. Gopikrishna and Parameswaran [ 29 ] indicated that premedication with 50 mg rofecoxib and 600 mg ibuprofen as single-dose significantly decreased post-endodontic pain at 4 and 8 hours compared with the placebo. However, at 12 and 24 hours, the pain score in rofecoxib group was significantly lower than that in the ibuprofen and placebo groups. Mokhtari *et al*. [ 14 ] demonstrated that ibuprofen and indomethacin alleviated post-endodontic pain at 8 hours after the procedure; still, no significant difference was found between the two intervention groups and the control group in pain score at 12 and 24 hours. Baradaran *et al*. [ 30 ] indicated a significant reduction in post-endodontic pain in groups that received a combination of ibuprofen and alprazolam at 4, 6, and 12 hours compared with the ibuprofen group alone and the placebo. On the other hand, no significant difference was found among the groups at 24, 48, and 72 hours. Nonetheless, a different study found no significant difference in pain score of patients with reversible pulpitis whom received 200 mg ibuprofen versus 200 mg ibuprofen plus 216.7 mg acetaminophen. Both groups had moderate to severe pain until day 2; but the pain intensity started to decrease from the second day in both groups [ 31 ]. Similar to the present study, in the abovementioned studies, NSAIDs such as diclofenac, indomethacin, or a combination of ibuprofen with rofecoxib caused pain relief after endodontic treatment. 

A worth mentioning finding of the present study was the ability of premedication with ibuprofen to decrease pain by up to 24 hours after the procedure while the majority of previous studies [ 9 , 14 , 29 ] reported its maximum duration of efficacy to be 8 hours postoperatively, which is reasonable considering the half-life of ibuprofen. All strong and effective analgesics need to be repeated due to short half-life [ 9 , 29 ]. The difference between the results of the abovementioned studies and the present findings may be attributed to different dosage, frequency of administration, and type of tooth undergoing endodontic treatment. Ibuprofen prevents pain and inflammation by inhibiting the cyclooxygenase enzyme. Cyclooxygenase has two forms of COX-1 and COX-2. COX-2 plays a role in the synthesis of prostaglandins, which are pain mediators. Inhibition of COX-2 can prevent prostaglandin synthesis, and minimize the stimulation of nociceptors and pain stimuli. Ibuprofen decreases pain and inflammation by inhibiting both cytoprotective COX-1 enzymes and inflammatory COX-2 enzymes [ 2 ]. In a systematic review and meta-analysis, Almuthhin *et al*. [ 4 ], indicated that corticosteroids and COX-2 inhibitors could control the post-endodontic pain for up to 12 hours after their administration. Nonetheless, NSAIDs and particularly ibuprofen indicated a high efficacy for pain reduction for up to 2 days after treatment [ 4 ]. Similarly, the present study presented significantly lower pain score in the intervention groups, compared with the placebo group, for up to 24 hours after endodontic treatment. 

In the present study, U. dioica significantly decreased VAS and WBFPS pain scores at 8, 12, and 24 hours after treatment, compared with the placebo. No study on the effect of U. dioica on post-endodontic pain has been conducted, however, some animal and human studies have been conducted on the analgesic efficacy of U. dioica for pain relief in other body parts [ 18 - 19 , 26 , 32 - 34 ]. Marrassini *et al*. [ 32 ] reported the antinociceptive and anti-inflammatory effects of the ethanolic extract of the aerial parts of Urtica urens (500 mg/kg oral dosage) on rats through a writhing test. They stated that chlorogenic acid was responsible for such effects. Consistent with the present results, a different study indicated the analgesic effects of U. dioica on rats [ 18 ]. Another study on Wistar rats demonstrated the analgesic effects of the hydroalcoholic extract of U. dioica on pain caused by acetic acid. The study indicated that the analgesic effect of U. dioica was significantly greater than that of diclofenac [ 19 ]. Another study found that the ethanolic extract of Urtica circularis in 100 mg/kg concentration had greater analgesic effects than indomethacin following administration of acetic acid [ 26 ]. Safari *et al*. [ 33 ] indicated the anti-inflammatory, anti-pyretic, and analgesic effects of U. dioica on rats, although diclofenac was more effective. In the abovementioned studies, the analgesic effects of U. dioica were superior to those of diclofenac and indomethacin NSAIDs. On the other hand, in the present study, no significant difference was noted in analgesic efficacy of premedication with U. dioica and ibuprofen for reduction of post-endodontic pain. Vatankhah *et al*. [ 34 ] reported significantly lower postoperative endodontic pain at 2, 4, and 24 hours in patients with symptomatic irreversible pulpitis who received diclofenac, compared with ibuprofen. Moreover, the aforementioned studies highlighted the superiority of the analgesic effects of U. dioica compared with diclofenac. Consequently, it was expected that U. dioica would have higher analgesic efficacy than ibuprofen; nevertheless, they did not have a significant difference in analgesic efficacy in the present study. The reason may be that the aforementioned studies were animal studies, and the method of pain measurement was more subjective than that in the present study. Moreover, the dosage and time of administration of medications, and the sample size were different from those in the present study, and this study was conducted under controlled conditions. Moreover, the severity of post-endodontic pain depends on several factors, such as the gender, tooth type, presence, and size of the periapical lesion, number of treatment sessions, extrusion of infected debris, application of irrigation solution into the periapical tissue, amount of extruded debris and instrument technique [ 35 ]. In the study by Agrawal *et al*. [ 7 ] the step-back preparation technique was correlated with lower pain intensity than the crown-down preparation, conventional instrumentation, and hybrid technique.

Unlike the abovementioned studies, Hajhashemi and Klooshani [ 18 ] indicated that leaf extract of U. dioica in 100, 200 and 400mg dosage decreased abdominal cramp pain by 41%, 64%, and 81% in rats; while, this rate was 84% by indomethacin. Evidence [ 21 , 33 ] displays the direct analgesic effects of U. dioica through blocking of nociceptors and inhibition of synthesis and release of inflammatory pain mediators such as prostaglandins. Correspondingly, the analgesic and anti-inflammatory effects of U. dioica are attributed to flavonoids, polyphenolic compounds, and triterpenes. The possible mechanism of action of U. dioica against inflammation is through plant phytochemicals such as flavonoids, phenolic acids, and tannins [ 21 , 36 ]. Likewise, Farahpour *et al*. [ 19 ], attributed the analgesic efficacy to the presence of flavonoids, caffeoyl malic acid, and the caffeic acid. It may be stated that in the present study, these factors most likely played a role in reduction of pain and inflammation. Another study evaluated the effect of U. dioica on clinical symptoms such as pain and paraclinical parameters of rheumatic patients, and showed the positive anti-inflammatory effects of U. dioica on such patients [ 37 ]. A meta-analysis pointed to the analgesic and anti-inflammatory effects of U. dioica on neural and muscular pains [ 15 ]. U. dioica has an inhibitory effect on nuclear factor kappa B, NF-kB activation, which is a regulator of pro-inflammatory cytokines, and can decrease neural and muscular pain by exerting analgesic and anti-inflammatory effects [ 15 ]. According to the results of the present study, the null hypothesis was accepted. 

Considering the comparable analgesic efficacy of U. dioica and ibuprofen, and other pharmaceutical and nutritional benefits of U. dioica [ 24 - 25 ] with no side effects, it may be suitable for pain relief in dentistry. Moreover, its safety is confirmed in several studies [ 38 - 39 ]. 

Considering the side effects of NSAIDs [ 40 ], further studies are required to find the optimal dosage of U. dioica for its widespread use. In addition, its antimicrobial effects should be investigated in future studies. Further studies with a larger sample size and longer follow-ups are required as well. Moreover, the effects of U. dioica on inflammatory factors need to be investigated. Since the VAS and WBFPS scores were found to be the same in the present study, use of VAS alone would suffice in future studies. 

The strength of the present study was that none of the participants was excluded from the study. Moreover, no side effects were observed. However, this study had some limitations. The pain score of patients at 8, 12 and 24 hours was self-reported by patients and with considering subjectivity of pain may not be highly accurate. The other limitation of this study was the small sample size.

## Conclusion

According to the present results, it seems that premedication with leaf extract of U. dioica or Netonal tablet is as effective as ibuprofen for reduction of post-endodontic pain in teeth with symptomatic irreversible pulpitis. Moreover, it preserves its analgesic efficacy for up to 24 hours after endodontic treatment.
